# Farmers’ Rural-To-Urban Migration, Influencing Factors and Development Framework: A Case Study of Sihe Village of Gansu, China

**DOI:** 10.3390/ijerph16050877

**Published:** 2019-03-10

**Authors:** Libang Ma, Meimei Chen, Xinglong Che, Fang Fang

**Affiliations:** Department of City and Resources, College of Geography and Environmental Science, Northwest Normal University, Lanzhou 730000, China; 15117173302@163.com (M.C.); chexinglon@163.com (X.C.); fangfangkim@163.com (F.F.)

**Keywords:** rural-to-urban migration, influencing factors, institutional analysis and development framework, Sihe village of Gansu province

## Abstract

Farmers are the major participants in rural development process and their willingness to settle in urban areas directly affects the implementation of rural revitalization strategy. Based on Ostrom’s institutional analysis and development (IAD) framework, we analyzed farmers’ willingness to settle in urban areas and its influencing factors by binary Logistic regression and cluster analysis of survey data of 190 rural households in Sihe village of Gansu Province of China. The results show that: (1) In Sihe village, farmers’ willingness to settle in urban areas was low in general and influenced by their neighbors’ decisions or behaviors. Households willing and unwilling to migrate to urban areas both presented significant spatial agglomeration. (2) The factors influencing farmers’ willingness to settle in urban areas were analyzed from six aspects: individual characteristics, family characteristics, residence characteristics, cognitive characteristics, institutions, and constraints. The main influencing factors were found to be age, occupation, number of non-agricultural workers in the family, household cultivated land area, annual household income, house building materials, degree of satisfaction with social pension, homestead and contracted land subsidies, income constraints, and other constraints. (3) Individual heterogeneity and difference in economic basis determined the difference in farmers’ willingness to settle in urban areas. Institutions and constraints played different roles in the migration willingness of different groups of farmers (Note: More details on the sample as well as further interpretation and discussion of the surveys are available in the associated research article (“Village-Scale Livelihood Change and the Response of Rural Settlement Land Use: Sihe Village of Tongwei County in Mid-Gansu Loess Hilly Region as an Example” (Ma, L.B.; Liu, S.C.; Niu, Y.W.; Chen, M.M., 2018)).

## 1. Introduction

The large-scale rural-to-urban migration in China since 1990s has been attracting the attention of researchers. Considering the actual situation of China and referring to classical migration theories, researchers have conducted extensive research on rural-to-urban migration in China [1,2,3,4]. At a macro-regional scale, some researchers, based on the “push–pull” hypothesis, have explained the influences of inter-regional forces on population migration and analyzed problems such as the return of migrant workers to rural areas, non-agricultural population changing to agricultural population, and farmers’ bird-like migration [5,6]. On this basis, the main influencing factors of population migration are discussed from regional structural aspects. At a micro-individual scale, neoclassical economics emphasizes the influence of subjective factors on population migration [7] and proposes that individuals will consider the costs and benefits of investment when making migration decisions. If the expected net income is positive, then individuals will transfer to urban areas. In this process, family is the decision maker, family members are the participants and their willingness to settle in urban areas is an important factor to promote urbanization [8,9,10,11]. In addition, migration network theory believes that the social ties between migrants and non-migrants based on consanguinity, kinship, and rural fellowship can reduce the cost and risk of farmers’ migration to urban areas and increase the expected benefits, thus encouraging farmers to migrate to urban areas [12].

In recent years, researchers have realized that the mechanism of rural-to-urban migration is very complicated and it is almost impossible to explain it by using only one theory. Therefore, it is necessary to combine the theories at both macro and micro scales to analyze population migration [13]. Relevant empirical research has begun to establish a multilevel theoretical framework. With the help of tools such as multilevel statistical models, researchers not only consider the independent roles of individual, family and regional factors in the migration of rural population, but also analyze the cross-level interactions of factors [14,15,16,17]. Especially, investigating the willingness of farmers is very fundamental and necessary [18].

The above-mentioned research enriches current understanding of farmers’ migration to urban areas, but there are also certain deficiencies. First, most of the research is based on the survey data of large-scale regions to analyze farmers’ willingness to settle in urban areas and its influencing factors, and there is lack of micro-scale analysis based on village units. Second, the research mainly focuses on the influences of individual and family characteristics on farmers’ willingness to migrate to urban areas, and the influences of institutions and constraints are ignored. In fact, farmers’ willingness to migrate to urban areas is not only subject to micro factors such as farmers’ behavior, family characteristics and cognitive ability, but also related to macro institutional factors such as policies, norms, and rules.

With China’s rapid industrialization, marketization and globalization as well as the adjustment of population policies, many rural laborers are able to migrate to towns and cities, which promotes urbanization in China [19,20,21,22,23]. However, during this process, there occur problems such as labor outflow, idle homestead and land grab in rural areas, which seriously affect the sustainable development of villages [24,25,26,27]. Moreover, problems such as shortage of infrastructure investment, poor industrial development, weak economic foundation, widening gap between rich and poor, human-land separation, population drift between urban and rural areas, and aging and declining rural communities are becoming more severe in rural areas. In 2017, “rural revitalization strategy” was first proposed in the Nineteenth National Congress of China to solve the above problems, release new energy for agricultural and rural development, and achieve sustainable rural development as well as urban–rural integration. Rural revitalization strategy is not only an important strategy to promote urban–rural integration and sustainable rural development, but also an inevitable requirement for solving the three rural issues and building a well-off society [28,29,30,31].

In recent years, we have carried out some research on the structure, pattern evolution, driving mechanism and layout optimization of rural settlements in the mid-Gansu loess hilly region [22,32,33,34]. It is found that the region is undergoing dramatic changes: the number of rural settlements is decreasing, village size is shrinking, the spatial distribution of rural settlements is becoming scattered, and rural settlement structure tends to be regular. Also, problems such as hollowing village and deteriorating rural environment are affecting rural revitalization [22,32,33,34]. One of the focuses of rural revitalization strategy is population. Excessive population loss in rural areas should be prevented and there should be a balance among people migrating to urban areas, staying in rural areas and returning to rural areas. The rural industry, environment and opportunities are supposed to attract people. Farmers are the major participants in rural development process and their willingness to settle in urban areas directly affects the implementation of rural revitalization strategy [35,36]. Therefore, analyzing farmers’ willingness to settle in urban areas and exploring its influencing factors are of great theoretical and practical significance for enriching the theory of rural-to-urban migration and promoting the implementation of rural revitalization strategy.

Farmers’ willingness to migrate to urban areas is not only affected by micro-factors such individual characteristics, family financial situation and cognitive ability, but also related to macro-factors such as institutions. However, current studies mainly consider the influences of individual and family characteristics on farmers’ willingness to migrate to urban areas and often fail to include policy environment into the research framework. Moreover, most of these studies are based on the survey data of large-scale regions and there is lack of micro-scale analysis based on village units. In this paper, Sihe village of Gansu Province was selected as the study region. Based on Ostrom’s institutional analysis and development (IAD) framework [37,38,39,40], we constructed an analysis framework that took macro institutional factors into account and identified the factors influencing farmers’ willingness to migrate to urban areas. On this basis, we further analyzed the influence of interaction between individual characteristics/family economic basis and institutions/constraints on farmers’ willingness to migrate to urban areas. Such an analysis can better reflect the actual situation in rural areas. The results can provide scientific support for solving the problem of “population” during the implementation of rural revitalization strategy.

## 2. Overview of Study Region

**Figure 1:**
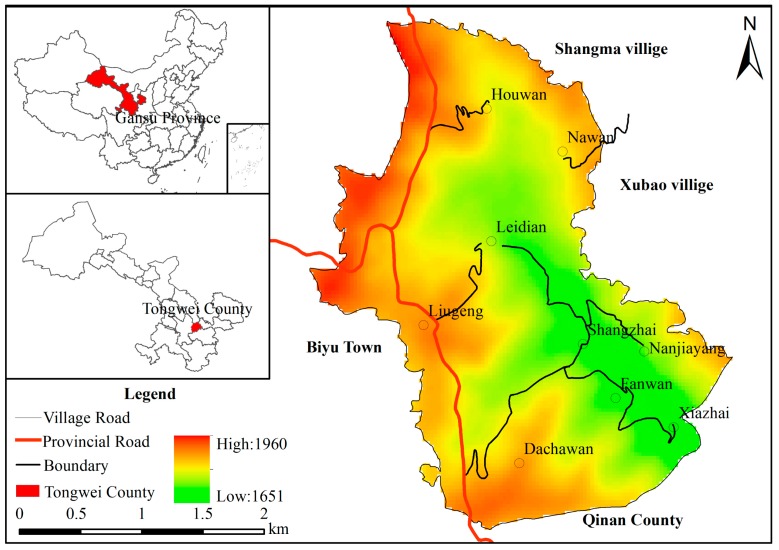
Survey map of the study region.

Sihe village is located in the mid-Gansu loess hilly region of China and specifically in the west part of Jichuan town in Tongwei county of Gansu Province (105°25′52.97″ E and 35°8′10.21″ N). It has a total area of 7.71 km^2^, 15 km distant from town area and 25 km distant from county seat. Sihe village is adjacent to Shangma village and Xubao village of Tongwei County in the northeast and adjacent to Wangfu Town of Qin’an County in the southwest (Figure 1). The village is about 1600–2000 m above sea level, with average altitude of 1763 m. It has a temperate and humid climate, with annual average precipitation of 450 mm, frost-free period of 150 days and adequate solar radiation [22].

Sihe village has jurisdiction over nine communities including Dachawan, Shangzhai, Leidian, Napowan, Houwan, Nanjiayang, Xiazhai, Fanwan, and Liugeng. Among them, Liugeng is located on the top of mountain, Shangzhai and Xiazhai are located in valley region and the remaining communities are distributed on mountain slopes. In 2017, there were a total of 327 households in Sihe village. The total population were 1233, among which 700 were labor force participants. The per capita net income was 3000 yuan and the income sources included crop farming, poultry and livestock raising, forestry and fruit industry, working outside of the village, and other forms of business. There were seven households relying on only one livelihood and more than 75% of households relied on at least three livelihoods. The total cultivation area was 452.13 hm^2^, with per capita cultivation area of 0.31 hm^2^. The total area of rural settlements was 10.23 hm^2^, accounting for only 1.33% of total area of the village. There are two reasons why Sihe villages was selected for investigation: (1) It is a typical poor village in Tongwei county. Driven by economic benefits, more and more farmers choose to work or even settle in urban areas, which has greatly affected rural revitalization. (2) The natural environment and development of rural society in Sihe village are typical in mid-Gansu loess hilly region. Therefore, the study of Sihe village can provide theoretical reference for the sustainable development of other rural areas in mid-Gansu loess hilly region [22].

## 3. Data Sources and Research Methods

### 3.1. Data Sources

Data came from three sources: (1) Basic maps: Topographic map of Sihe village (1:50,000) and vector administrative boundary (1:50,000) were obtained from Gansu Province Surveying and Mapping Bureau. (2) Spatial vector data of rural settlements: Image data were obtained by UAV aerial photography in 2017 and rural settlement patches were then extracted. (3) Field investigation. With the help of village head and community leaders of Sihe village, a 16-day household survey based on participatory rural appraisal (PRA) was conducted in July and December of 2017, with the survey in December as a supplementary survey. Interviews were conducted after we introduced ourselves, explained our purpose and gained the residents’ understanding and support. During the interview, information about their family, income and expenses, residence, production, behavior willingness, etc. were obtained. A total of 190 effective questionnaires were obtained, meaning that 76.92% of households in the village gave effective responses (80 households had already moved out of Sihe village and 57 households refused the interview, see Table 1). In addition, we interviewed some special villagers: villagers older than 65 years, village leaders who had worked for many years and did not retire, retired village leaders and accountants. For the reliability of data, at least two people were selected for each type and a total of 23 people were selected for all the types. The overall situation of the village was known through insider interview. The interview contents included the distribution and ownership of various land types; related land use policies; the main economic activities in the village; the internal structure, building material, morphology and function of rural houses in different periods; and farmers’ investment in their houses [22].

### 3.2. IAD Extension Decision Model

**Figure 2:**
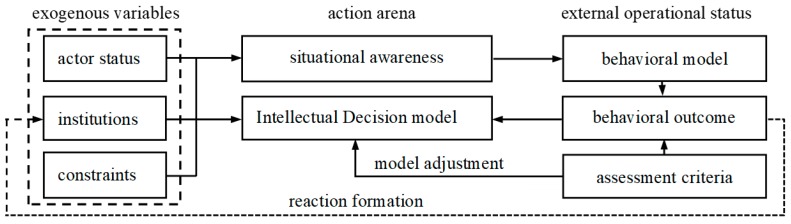
Institutional analysis and development (IAD) framework of farmers’ willingness to migrate to urban areas.

Traditional rational choice institutionalism assumes that an individual’s actions are completely determined by the rational calculation under the judgment of personal preference maximization. Since it fails to take into account the influence of institutional environment, actors are only regarded as atomized individuals without social connotation [41,42]. Therefore, rational choice institutionalism has been widely criticized. In this background, the Indiana School, represented by American scholar Ostrom, relaxed the hypothesis of humanity in traditional rational choice institutionalism. Although rational individual remains the logical starting point for research, institutional situation is included into individual’s strategic choice and then researchers examine how individuals maximize their benefits under the constraint of institutional situation. The modified rational choice institutionalism was finally developed by Ostrom into institutional analysis and development (IAD) framework for the study of interaction between individual choice and institutional situation (Figure 2) [43]. IAD framework integrate many disciplines and has multiple origins. Its theoretical basis includes classical political economics, neoclassical microeconomics, public choice theory, transaction cost economics and non-cooperative game theory. IAD framework can be used to study not only static institutional arrangements, but also the dynamic institutional arrangements of new rules and new technologies. It decomposes individual’s economic behavior into several interrelated and mutually restrained components, thus it not only enables detailed analysis of specific issues, but also can take various issues into a comprehensive consideration [44].

According to IAD framework, the rational choice of individual is influenced by five variables: the attributes of physical world, the community attributes of the field in which the action takes place, the rules that motivate the behavior, action situation, and individual’s psychological activity. Among them, the first three are exogenous variables and the remaining two are endogenous variables. Exogenous variables include all aspects of social, cultural, institutional, and material environments that the participants are in. Especially, the action situation determines the internal mechanism of decision-making of actors and directly affects the structure of behavior. It is also the core component of the framework and can be divided into two parts: action arena and external action situation. It determines how an individual interacts with exogenous variables and behaves in the whole institutional framework. In the internal structure of the action situation, individuals observe the changes of environmental information and then take actions. Therefore, the outcome is determined by the action pattern and exogenous variables. The outcome has feedback effect on the components of IAD framework. This is triggered by participants’ evaluation of actions and outcomes based on the information they can observe and process [45]. In addition, rules are the most operable set of exogenous variables directly affecting action scenarios in the IAD framework. They are instructions on how to establish action scenarios in a specific environment, strategies for constructing action scenarios recognized by participants, or efforts to maintain order and predictability of situations in view of the association between actions and outcomes. In short, rules play a central role in action situations. Ostrom divides rules into seven categories including identity rules, boundary rules, selection rules, aggregation rules, scope rules, information rules and reimbursement rules. Some categories of the rules may not exist and the lack of one or more rules may lead to corresponding default variables under no direct rule constraints and then affect other variables. If there are no variables in an action scenario, then the action scenario is in Hobbes’s natural state, which will inevitably lead to the chaos of the system and participants’ behavior. Ostrom argues that commitment to institutional reform often involves the formulation or adjustment of rules affecting action scenarios [46].

IAD framework has been improved with Ostrom’s long-term efforts. Especially, its applicability and explanatory power have been significantly improved. The relationship between internal structure of action situation and rules has become clearer and has been supported by increasing cases. For example, the framework has been widely applied to planting, requisition of land and cultivated land recuperation in rural areas [47,48,49]. IAD framework takes natural material conditions and community attributes into account, guides researchers to clarify the relationship between resources and occupiers through a series of application rules, and constantly improves the self-governance system in practice [50].

Farmers’ willingness to migrate to urban areas in various stages of socioeconomic development is influenced by factors at both micro and macro scales. At a micro scale, the individual and cognitive characteristics of farmers are the most fundamental factors influencing their willingness to migrate to urban areas. At the same time, the family and residence characteristics of farmers can influence their cognition. Currently, the living conditions in urban areas are better than those in rural areas, thus migration to urban areas has become an optimum choice for farmers in terms of settlement. At a macro scale, factors influencing farmers’ willingness to migrate to urban areas include not only the physical environment and community attributes, but also institutional structure or rules. The difference in physical environment between rural and urban areas can influence farmers’ residential preference. When their financial ability reaches a certain level, rural residents tend to migrate to urban areas. The better living and production environment in urban areas also attracts rural residents to settle in these areas. In sum, farmers’ migration to urban areas is motivated and constrained by many factors, which play roles in increasing or decreasing farmers’ willingness to settle in urban areas. On above basis, IAD framework was used here to include multilevel variables that can influence farmers’ willingness to migrate to urban areas and a research framework for studying the mechanism of farmers’ rural-to-urban migration was proposed. In this framework, farmers’ migration willingness is not only affected by micro factors (individual characteristics, family characteristics, residence characteristics and cognitive characteristics), but also affected by macro factors (institutions).

The components of the research framework are interrelated and mutually restricted. Farmers’ perception of information is influenced by exogenous variables such as their status (individual characteristics, family characteristics and residence characteristics), institutions (government encouragement and homestead and contracted land policy), and constraints (income, employment, housing, social security, and other constraints). The migration behavior of farmers who have already moved to urban areas also has an effect on the decision-making process of farmers who have not yet migrated to urban areas. Especially, the degree of difference in living standards and income levels between them affects the decision-making of the latter. In other words, the behavior of some farmers will affect the decision-making or migration behavior of others.

### 3.3. Logistic Model

(1) Model Construction

The willingness of farmers to migrate to urban areas is a random-choice problem. In the questionnaire, four options were designed for the problem, namely, unwillingness = 0, willingness = 1, uncertainty = 2, and no consideration = 3. In this paper, the explained variable was the willingness of farmers to settle in urban areas, which belongs to a 0–1 variable. Logistic model was used for analysis. This model is a binary discrete choice model that uses logical distribution as the probability distribution of random error terms. It is suitable for the analysis of selection behavior based on the principle of utility maximization. It is the most ideal and widely used model in analyzing the decision-making behaviors of individuals. On this basis, cases including uncertainty = 2 and no consideration = 3 were not included into our study. It was defined that dependent variable “y = 1” indicates farmers are willing to migrate to urban areas and “y = 0” indicates farmers are unwilling to migrate to urban areas. Independent variable x is the variable that could influence farmers’ willingness to migrate to urban areas. Suppose that $f\left(x\right)=\beta_{0}+\beta_{1}x_{1}+\beta_{2}x_{2}+\ldots +\beta_{n}x_{n}$ is a linear function for variables influencing farmers’ willingness to migrate to urban areas, then the probability that farmers are willing to migrate to urban areas is $P_{i}=e^{f\left(x\right)}/\left[1+e^{f\left(x\right)}\right]$ and the probability that farmers are unwilling to migrate to urban areas is $1-P_{i}$. By logarithmic conversion, we can obtain $ln\left(\frac{P_{i}}{1-P_{i}}\right)=f\left(x\right)$. The specific model is as follows:

(1) $$P_{i}=F\left(\alpha +\overset{m}{\underset{j=1}{\sum }}\beta_{j}x_{ij}\right)=1/\left(-\alpha +\overset{m}{\underset{j=1}{\sum }}\beta_{j}x_{ij}\right)$$

where *P_i_* is the probability that the *i*th farmer is willing to migrate to urban areas; *β_j_* is the regression coefficient of the *j*th influencing factor; *m* is the number of influencing factors; *x_ij_* is independent variable, indicating the *j*th influencing factor of *i*th farmer; α is regression intercept.

(2) Selection and Definition of Variables

Farmers’ migration to urban areas is an event with varying possibilities. It is not the choice of one individual but the decision of the whole family after comprehensive consideration of individual and family situation as well as external environment. Following the principles of representativeness, comparability and quantifiability and in accordance with relevant literatures, 28 variables were selected from 6 aspects as factors influencing farmers’ willingness to migrate to urban areas (Table 2)

## 4. Results

### 4.1. Farmers’ Willingness to Migrate to Urban Areas

#### 4.1.1. Overall Patterns of Farmers’ Willingness to Migrate to Urban Areas

**Figure 3:**
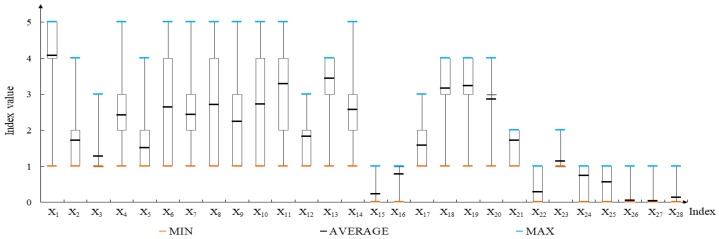
Statistics of the independent variables influencing farmers′ willingness to migrate to urban areas.

Among the 190 interviewed rural households, 26% (50 households) had the willingness to migrate to urban areas, 56% (107) had no willingness to migrate to urban areas, 5% (9) were uncertain about migration to urban areas and 13% (24) had never considered about migration to urban areas (Figure 3). Overall, there was a relatively low willingness of residents in Sihe village to migrate to urban areas. In addition, the annual average income of households that were willing to migrate to urban areas reached 57,600 yuan and their non-agricultural income reached 42,800 yuan. Among these farmers, 74% were younger than 60 years, 50% had middle school degree or higher, 12% had a high economic level, 32% had the ability to purchase a commercial house in urban areas, and 30% were willing to give homestead and contracted lands back to the village collective. With such capitals, these farmers and their families were willing to settle in urban areas that could provide more job opportunities and better living conditions (Figure 3).

#### 4.1.2. Spatial Patterns of Farmers’ Willingness to Migrate to Urban Areas

**Figure 4:**
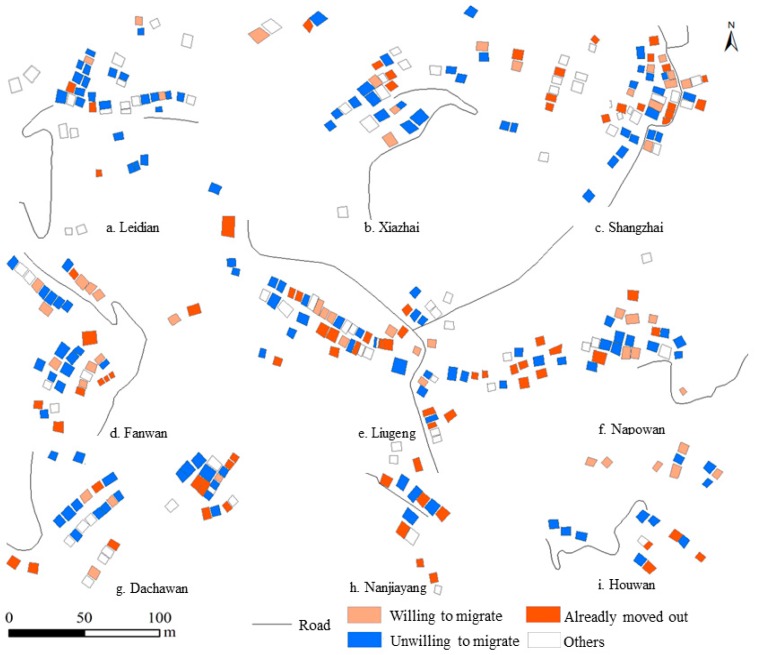
Distribution of households with different attitudes towards migration to urban areas in the nine communities of Sihe village.

The willingness of farmers to migrate to urban areas was affected by the decisions or behaviors of neighboring residents. During investigation, it was found that farmers with kinship often lived close to each other and had similar residential behavior. If one family had already settled in urban areas, then their relatives often had strong willingness to migrate to urban areas. This phenomenon was very significant in Houwan, Fanwan, Liugeng and Shangzhai (Figure 4). Compared with other communities, these four communities were characterized by the highest proportion of households that had the willingness to migrate to urban areas or already migrated to urban areas, reaching 47.62%, 43.24%, 43.21%, and 41.07%, respectively.

Households with no migration willingness also showed spatial agglomeration (strip-shaped or clustered distribution) and this phenomenon was observed in Leidian, Xiazhai, and Dachawan. These households mainly relied on agricultural production activities, and they were rarely engaged in non-agricultural production. Therefore, their sources of income were mostly limited to the agricultural production activities on their contracted lands. If there were not external stimuli such as new policies, the production and living needs of these households would still be satisfied through traditional family farming. In addition, these households were satisfied with their current production and living status and doubted whether they could live a better life in urban areas. Therefore, they preferred to stay in rural areas. Moreover, they were influenced by the behaviors of their neighbors. Since most of their neighbors were unwilling to migrate to urban areas and stayed in Sihe village, they were also unwilling to migrate to urban areas. In sum, farmers’ migration to urban areas was not only determined by themselves but also determined by many external factors.

### 4.2. Factors Influencing Farmers’ Willingness to Migrate to Urban Areas

#### 4.2.1. Benchmark Regression

This paper took farmers’ willingness to migrate to urban areas as explained variable and farmers’ individual characteristics, family characteristics, residence characteristics and cognitive characteristics as explanatory variables. Binary Logistic regression analysis of these variables was performed using SPSS20.0 software (model 1). In addition, institutions and constraints were included as another two explanatory variables and Logistic regression analysis was performed again (model 2). On this basis, their influence on farmers’ willingness to migrate to urban areas was analyzed (Table 3). The Cox & Snell *R*^2^ and Nagelkerke *R*^2^ in model 2 increased by 101.9% and 93.5% compared with those in model 1, respectively. This illustrated that institutions and constraints had important influence on famers’ willingness to migrate to urban areas. Therefore, model 2 was mainly discussed here.

#### 4.2.2. Factors Influencing Farmers’ Willingness to Migrate to Urban Areas

**Figure 5:**
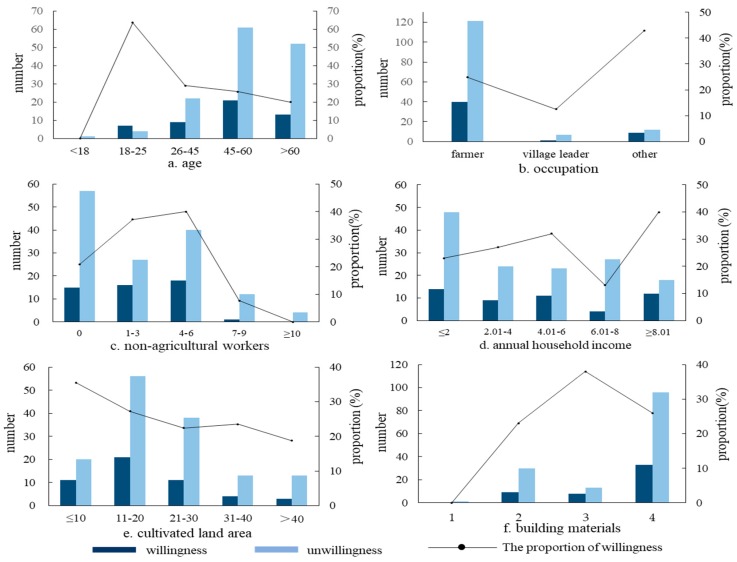
Farmers’ willingness to migrate to urban areas influenced by various factors. Note: In f, 1-adobes; 2-brick-wood structure; 3-brick-concrete structure; 4-steel-concrete structure.

In terms of individual characteristics, age and occupation influenced farmers’ willingness to migrate to urban areas at the 0.1 and 0.05 levels of significance, respectively. The interviewed farmers older than 18 years showed decreased willingness to migrate to urban areas with increase in age (Figure 5a). Among the farmers older than 60 years, only 20% planned to settle in urban areas. These old farmers had lived in Sihe village for a long time and had relatively low requirements for quality of life. They thought that they could not find a stable job with good pay in urban areas. Therefore, these old farmers preferred to live in the village. In addition, the migration willingness of farmers relying on agricultural production as livelihoods was 9.66% lower than that of farmers relying on both agricultural and non-agricultural production as livelihoods (Figure 5b). In other words, the latter were more willing to migrate to urban areas. This was because most of them worked in urban areas, accumulated related life experience and certain material capitals, and had a relatively broader horizon, which laid a foundation for their settlement in urban areas [17]. Village leaders often had a relatively stable income in addition to the income from agricultural production and the former was even higher than the latter. Therefore, village leaders were unwilling to give up their stable wage work and to migrate to urban areas.

In terms of family characteristics, the number of non-agricultural workers in the family and cultivated land area negatively influenced farmers’ willingness to migrate to urban areas at the 0.05 level of significance, whereas family annual income positively influenced farmers’ willingness to migrate to urban areas at the 0.1 level of significance. The 173 interviewed households with less than 7 non-agricultural workers showed increased willingness to migrate to urban areas with increase in the number of non-agricultural workers (Figure 5c). When the number of non-agricultural workers exceeded seven, however, the willingness to migrate to urban areas decreased. Generally, the greater the number of non-agricultural workers, the better the financial situation of a family and the stronger the willingness to migrate to urban areas. It was found that all laborers in families with more than seven non-agricultural workers indeed worked in urban areas. The interviewed farmers, as representatives of their families, were the old members in these families and, influenced by traditional lifestyles, they were unwilling to migrate to urban areas. With increase of annual income and improvement of financial situation of family, the willingness to migrate to urban areas increased (Figure 5d). Settling in urban areas is a permanent migration with high investment cost and only families with good financial situation can afford it [17]. The larger the cultivated land area of a family, the lower the willingness to migrate to urban areas (Figure 5e). This was closely related to the various agricultural policies implemented in recent years. Although the proportion of agricultural income in net income of farmers was decreasing, most farmers, influenced by traditional values, still hoped to get various agricultural subsidies from the government and did not want to lose their lands because of migration to urban areas, showing strong land dependence.

In terms of residence characteristics, building materials negatively influenced farmers’ willingness to migrate to urban areas at the 0.1 level of significance. With the improvement of materials used for building houses, farmers’ willingness to migrate to urban areas increased first and then decreased (Figure 5f). The use of adobes as building materials suggested relatively low income levels and these families could not afford the high cost of living in urban areas. The use of bricks, woods, and concrete as building materials suggested relatively high income levels and good living conditions. Settling in urban areas meant that they had to buy a house in urban areas, which would cost a large amount of money. Since these families were satisfied with their current living environment in the village, they were unwilling to migrate to urban areas.

In terms of cognitive characteristics, degree of satisfaction with social pension negatively influenced farmers’ willingness to migrate to urban areas at the 0.01 level of significance. The higher the degree of satisfaction with current social pension, the lower the willingness to migrate to urban areas. This illustrated that farmers were relatively satisfied with the current pension policy of the country and had good expectations for future rural pension policy. They believed that living in rural areas also enabled a good pension security, thus they were reluctant to migrate to urban areas.

In terms of institutions, homestead and contracted land subsidies positively influenced farmers’ willingness to migrate to urban areas at the 0.01 level of significance. About 85.2% of the households interviewed wanted to retain their homesteads and contracted lands, and the remaining 14.8% wanted to give them back to the village collective. Among them, the proportions of rural households willing to migrate to urban areas were 21.7% and 53.6%, respectively. Although only a few farmers were willing to give homestead and contracted lands back to the village collective, the proportion of farmers willing to migrate to urban areas in them was high.

In terms of constraints, income constraint and other constraints negatively influenced farmers’ willingness to migrate to urban areas at the 0.01 and 0.05 levels of significance, respectively. For most farmers, migration to urban areas was a decision that would cost a large amount of money. If farmers did not have a certain amount of funds or stable income, most of them would choose to stay in the village. Therefore, income was a decisive factor for the migration of farmers to urban areas. In terms of other constraints, many farmers said that they were accustomed to rural life and thought that urban life was inconvenient and characterized by high consumption levels. They were afraid that they could not adapt to the urban life or find a stable job with good pay. Therefore, they wanted to stay in rural areas.

### 4.3. Cluster Analysis of Farmers’ Willingness to Migrate to Urban Areas

IAD extension decision model decomposed farmers’ willingness to migrate to urban areas into several interacting components. The above-mentioned exogenous variables affected the decision-making process of farmers, and the results would in turn directly or indirectly affect the exogenous variables. As the financial requirements for migration to urban areas were getting higher and higher, the primary premise for migration to urban areas should be the personal preferences or behavior patterns and financial ability of farmers. Therefore, individual characteristics and economic basis would affect farmers’ willingness to migrate to urban areas. In this paper, cluster analysis was performed to study the roles of homestead and contracted land subsidy policy, income constraint and other constraints in the migration willingness of different groups of farmers classified according to individual characteristics and family economic basis.

#### 4.3.1. Farmers’ Individual Characteristics

According to the age, education level and occupation of interviewed farmers, they were divided into youth group (<45 years) and middle-aged/aged group (≥45 years); high education group (high school or above) and low education group (below high school); agricultural production group (mainly engaged in agricultural production activities) and non-agricultural production group (mainly engaged in non-agricultural production activities). Cluster analysis results revealed the different roles of institutions (homestead and contracted land subsidy policy) and constraints (income constraint and other constraints) in the migration willingness of different groups of farmers (Table 4).

The influence of homestead and contracted land subsidy policy on farmers’ willingness to migrate to urban areas was greater in the youth group than in the middle-aged/aged group. In other words, there was a higher proportion of farmers willing to migrate to urban areas in the former group than in the latter group under the influence of the policy. In the youth group, many farmers were willing to give their homestead and contracted lands back to the village collective and then get subsidies to increase their capitals for migration to urban areas. With increase in age, the influence of constraints became stronger. The increase in age reduced the human capital of farmers, making it difficult for them to find suitable jobs in urban areas and to adapt to urban life. However, if they could find stable jobs with good pay in urban areas, they would become more willing to migrate to urban areas.

The influence of institutions and constraints was stronger in low education group than in high education group. Farmers with lower level of education had fewer opportunities to find a job in urban areas, thus they were more dependent on institutions to support them. Moreover, these farmers generally focused more attention on various constraints, and low income level and adaptation to urban life were two constraints for their migration to urban areas.

The influence of homestead and contracted land subsidy policy on farmers’ willingness to migrate to urban areas was greater in the agricultural production group than in the non-agricultural production group. The farmers in the former group were highly dependent on their lands. If without necessary guarantee, they would not easily give up their lands and migrate to urban areas. The influence of constraints on farmers’ willingness to migrate to urban areas was greater in the non-agricultural production group than in the agricultural production group. For the former group, if their life and work in urban areas could be ensured, they would become more willing to migrate to urban areas.

#### 4.3.2. Rural Family Economic Basis

According to the number of non-agricultural workers in the family and family annual income, farmers were divided into group without non-agricultural workers (non-agricultural workers = 0) and group with non-agricultural workers (non-agricultural workers > 0); low income group (family annual income < 50,000 yuan) and high income group (family annual income ≥ 50,000 yuan). Cluster analysis results revealed the different roles of institutions and constraints in the migration willingness of different groups of farmers (Table 5).

The influence of institutions was greater in the group without non-agricultural workers and low income group than in the group with non-agricultural workers and high income group, respectively. Lands were the only source of income for low-income families without non-agricultural workers and a guarantee for their life and production. Therefore, they were unwilling to give their lands back to the village collective and to migrate to urban areas. Their willingness to migrate to urban areas was greatly influenced by institutions. The influence of constraints was greater in the group with non-agricultural workers and high income group than in the group without non-agricultural workers and low income group, respectively. The income sources of the former two groups were more diversified, and their incomes were relatively high. Some of the family members in these two groups had already lived and worked in urban areas. Therefore, they were more concerned with the economic conditions in urban areas and constraints had a greater impact on them.

## 5. Discussion and Conclusions

### 5.1. Discussion

With emphasis put on the coordinated development of urban and rural areas, China has successively implemented a series of macro strategies such as coordinated urban and rural development, new rural construction, urban–rural integration, and new urbanization. [51]. On this basis, China further proposed rural revitalization strategy [52], with the aim of solving problems such as unbalanced development of urban and rural areas and inadequate rural development and helping to build a well-off society [21]. However, the urban–rural dual system characterized by long-term urban–rural separation and high-priority urban development have caused the transfer of rural labor force, resources, capitals and other production factors to urban areas, which restrict the sustainable development of rural areas [52]. This has also caused increasingly severe “village diseases”, one of which is high-speed transformation of production factors such as lands and population [51]. The rapid urbanization in China has resulted in an annual loss of nearly 200 thousand hectare of cultivated lands in rural areas, more than 100 million farmers who lose their lands, 290 million migrant workers who left their villages and worked in urban areas, human-land separation, population drift between urban and rural areas, and transfer of rural youth labor force to non-agricultural sector. These have intensified the problem of “three leftover populations” and aging population in rural areas. Population and labor force decrease in rural areas limit the development of modern agriculture and rural transformation [3]. Therefore, in order to realize rural revitalization, the problem of “population” should be solved first.

Farmers’ willingness to settle in urban areas directly affects the future development of rural areas. In Sihe village, about 24.5% (80) of households had already migrated to urban areas. Individual heterogeneity and difference in economic basis lead to difference in farmers’ willingness to migrate to urban areas. Overall, there was a relatively low willingness of farmers in Sihe village to migrate to urban areas. However, once certain conditions are achieved, there will be many farmers who choose to settle in urban areas, which will pose a great challenge to future rural development. In order to realize the revitalization of villages such as Sihe village, it is necessary to first encourage people to stay in the village and then attract more people to the village. This means government should encourage people to start businesses in the countryside and guide farmers to return to their hometowns to participate in production activities [25].

The IAD framework was adopted here to analyze farmers’ willingness to settle in urban areas at a village scale and its influencing factors. This paper took into account, for the first time, macro institutional factors to expand existing research on farmers’ willingness to migrate to urban areas. Our work provides not only a new method, but also a new perspective for studying farmers’ willingness to migrate to urban areas. However, the study area and sample size are small. Further research on areas with different terrains and economic development levels is needed, which can help solve the problem of “population” and achieve rural revitalization. In addition, this is just a preliminary work and Sihe village is the first village that we chose for study. In the future, we will further improve the questionnaire, supplement some key variables, and choose at least 30% of the villages in the mid-Gansu loess hilly region of China for investigation. On such basis, an in-depth and comparative study will be conducted on the willingness of farmers to migrate to urban areas and their attitudes towards land expropriation in villages with different rural settlement structures and patterns. Besides, a 5- or 10-year follow-up survey of villages is necessary and then comparative time-series analysis of villages can be conducted.

### 5.2. Conclusions

IAD extension decision model was used to analyze the factors influencing farmers’ willingness to migrate to urban areas from six aspects: individual characteristics, family characteristics, residence characteristics, cognitive characteristics, institutions, and constraints. By binary Logistic regression and cluster analysis of questionnaire data of 190 rural households in Sihe village, we obtained the following conclusions:

(1) Among the 190 interviewed rural households, 26% (50 households) had the willingness to migrate to urban areas, 56% (107) had no willingness to migrate to urban areas, 5% (9) were uncertain about migration to urban areas and 13% (24) had never considered about migration to urban areas. Overall, there was a relatively low willingness of residents in Sihe village to migrate to urban areas. The willingness of farmers to migrate to urban areas was found to be affected by the decisions or behaviors of neighboring residents. Households willing and unwilling to migrate to urban areas both presented significant spatial agglomeration.
(2) The factors influencing farmers’ willingness to migrate to urban areas involved six aspects: individual characteristics, family characteristics, residence characteristics, cognitive characteristics, institutions, and constraints. The main factors influencing farmers’ willingness to migrate to urban areas included age, occupation, number of non-agricultural workers in the family, cultivated land area, family annual income, house building materials, degree of satisfaction with social pension, homestead and contracted land subsidies, income constraint, and other constraints. Among them, occupation, family annual income, house building materials, and homestead and contracted land subsidies had positive influence on farmers’ willingness to migrate to urban areas, whereas the other factors had negative influence on farmers’ willingness to migrate to urban areas.
(3) Individual heterogeneity and difference in economic basis lead to difference in farmers’ willingness to migrate to urban areas. Institutions and constraints played different rules in the migration willingness of different groups of farmers. The influence of institutions on farmers’ willingness to migrate to urban areas was greater in the youth group (versus middle-aged/aged group), the low education group (versus high education group), the agricultural production group (versus non-agricultural group), the group without non-agricultural workers (versus group with non-agricultural workers) and the low income group (versus high income group). The influence of constraints on farmers’ willingness to migrate to urban areas was greater in the middle-aged/aged group, the low education group, the non-agricultural production group, the group with non-agricultural workers and the high income group.
(4) Through the analysis of farmers’ willingness to migrate to urban areas and its influencing factors, some policy insights can be obtained to promote the implementation of new urbanization and rural revitalization strategies. First, government should create conditions for farmers to start their own businesses or find jobs in rural areas. Farmers play an important role in rural revitalization. Therefore, great attention should be paid to current farmers in rural areas and migrant workers that possibly return to rural areas. Combining with local economic development, government may provide financial support, tax relief, technical training and intermediary services to farmers. Second, government should take measures to improve the education level of framers. Farmers’ education level plays an important role in their migration to urban areas. Therefore, government should increase investment in rural continuing education, improve farmers’ education levels, and increase their human capitals. Third, government should establish a mechanism to protect the rights and interests of farmers who withdraw from the homestead and contracted land system. The current rural land management system should be further improved, farmers’ withdrawal from homestead and contracted land system should standardized, and government should enhance farmers’ political participation and create conditions for them to participate in the reform and innovation of rural land management system. Fourth, government should improve the basic education conditions in rural areas. Specifically, government should improve the education management system in rural areas, increase investment in the construction of necessary infrastructures in rural primary and secondary schools, improve the status and salary of rural teachers, advocate modern teaching methods, fundamentally improve the level of rural educational facilities, and finally promote urban–rural equivalence.

## Figures and Tables

**Table 1:** Household survey of Sihe village.

Community Name	Number of Total Households	Number of Surveyed Households	Ratio of Surveyed Households to Total Households (%)	Number of Unsurveyed Households	Number of Households That Moved Out of Village
Dachawan	38	22	57.89	6	10
Shangzhai	47	24	51.06	14	9
Xiazhai	34	27	79.41	3	4
Fanwan	34	24	70.59	3	7
Leidian	38	25	65.79	10	3
Napowan	21	15	71.43	1	5
Nanjiayang	14	4	28.57	2	8
Liugeng	75	36	48.00	17	22
Houwan	26	13	50.00	1	12
Sihe village	327	190	58.10	57	80

**Table 2:** Selection of factors influencing farmers’ willingness to migrate to urban areas and value assignment.

Variables	Value Assignment
(1) Individual characteristics	
Age (*X*_1_)	<18 years = 1; 18–25 years = 2; 26–45 years = 3; 46–60 years = 4; >60 years = 5.
Education level (*X*_2_)	Primary school or below = 1; middle school = 2; high school or technical secondary school = 3; college or above = 4.
Occupation (*X*_3_)	Farmer = 1; village leader = 2; others = 3.
(2) Family characteristics	
Family size (*X*_4_)	≤3 = 1; 4–6 = 2; 7–9 = 3; >9 = 4.
Number of non-agricultural workers (*X*_5_)	0 worker = 1; 1–3 workers = 2; 4–6 workers = 3; 7–9 workers = 4; ≥10 workers = 5.
Proportion of non-agricultural workers (*X*_6_)	≤20% = 1; 21–40% = 2; 41–60% = 3; 61–80% = 4; 81–100% = 5.
Cultivated land area (*X*_7_)	≤0.65 ha = 1; 0.67–1.33 ha = 2; 1.34–2 ha = 3; 2.01–2.67 ha = 4; >2.67 ha = 5.
Family annual income (*X*_8_)	≤20,000 = 1; 20,000–40,000 = 2; 40,000–60,000 = 3; 60,000–80,000 = 4; >80, 000 = 5.
Family non-agricultural income (*X*_9_)	≤20,000 = 1; 20,000–40,000 = 2; 40,000–60,000 = 3; 60,000–80,000 = 4; >80, 000 = 5.
Family annual expenditure (*X*_10_)	≤10,000 = 1; 10,000–20,000 = 2; 30,000–40,000 = 3; >40,000 = 5.
Economic level (*X*_11_)	Low = 1; relatively low = 2; medium = 3; relatively high = 4; high = 5.
(3) Residence characteristics	
House construction time (*X*_12_)	2009–2017 = 1; 1999–2008 = 2; before 1998 = 3.
Building materials (*X*_13_)	Adobe bricks = 1; brick-wood structure = 2; brick-concrete structure = 3; steel-concrete structure = 4.
Per capita homestead area (*X*_14_)	≤ 30 m^2^ = 1; 31–60 m^2^ = 2; 61–90 m^2^ = 3; ≥ 91 m^2^ = 4.
(4) Cognitive characteristics	
Ability to purchase commercial house (*X*_15_)	Yes = 0; no = 1.
Residence trend (*X*_16_)	Scattered = 0; cluttered = 1.
Degree of satisfaction with neighborhood relationship (*X*_17_)	Very dissatisfied = 1; dissatisfied = 2; basically satisfied = 3; satisfied = 4.
Degree of satisfaction with living conditions (*X*_18_)	Very dissatisfied = 1; dissatisfied = 2; basically satisfied = 3; satisfied = 4.
Degree of satisfaction with social pension (*X*_19_)	Very dissatisfied = 1; dissatisfied = 2; basically satisfied = 3; satisfied = 4.
Degree of satisfaction with income (*X*_20_)	Very dissatisfied = 1; dissatisfied = 2; basically satisfied = 3; satisfied = 4.
(5) Institutions	
Policies that encourage migration to urban areas (*X*_21_)	Know these policies = 1; Do not know these policies = 0.
“One family, one house” policy (*X*_22_)	Know this policy = 1; Do not know this policy = 0.
Homestead and contracted land subsidy policy (*X*_23_)	Know this policy = 1; Do not know this policy = 0.
(6) Constraints	
Income constraint (*X*_24_)	Yes = 1; no = 0.
Employment constraint (*X*_25_)	Yes = 1; no = 0.
Housing constraint (*X*_26_)	Yes = 1; no = 0.
Social security (*X*_27_)	Yes = 1; no = 0.
Other constraints (*X*_28_)	Yes = 1; no = 0.

**Table 3:** Logistic regression analysis results of factors influencing farmers’ willingness to migrate to urban areas.

Variables		Model 1		Model 2	
		B	Sig.	B	Sig.
(1) Individual characteristics	Age (*X*_1_)	−0.369	0.09	−0.43	0.08
	Education level (*X*_2_)	−0.017	0.951	−0.015	0.962
	Occupation (*X*_3_)	0.243	0.502	0.473	0.05
(2) Family characteristics	Family size (*X*_4_)	−0.078	0.466	−0.003	0.976
	Number of non-agricultural workers (*X*_5_)	−0.237	0.229	−0.368	0.04
	Proportion of non-agricultural workers (*X*_6_)	−0.129	0.787	−0.25	0.623
	Cultivated land area (*X*_7_)	−0.029	0.09	−0.037	0.036
	Family annual income (*X*_8_)	0.206	0.219	0.246	0.09
	Family non-agricultural income (*X*_9_)	−0.158	0.356	−0.189	0.322
	Family annual expenditure (*X*_10_)	0.042	0.712	0.104	0.392
	Economic level (*X*_11_)	−0.207	0.362	−0.194	0.421
(3) Residence characteristics	House construction time (*X*_12_)	0.362	0.08	0.388	0.135
	Building materials (*X*_13_)	−0.167	0.418	0.439	0.091
	Per capita homestead area (*X*_14_)	0.003	0.603	0.004	0.495
(4) Cognitive characteristics	Ability to purchase a commercial house (*X*_15_)	0.477	0.264	0.277	0.544
	Residence trend (*X*_16_)	0.385	0.432	0.292	0.575
	Degree of satisfaction with neighborhood relationship (*X*_17_)	−0.112	0.751	−0.11	0.774
	Degree of satisfaction with living conditions (*X*_18_)	0.155	0.646	0.177	0.612
	Degree of satisfaction with social pension (*X*_19_)	−0.612	0.037	−0.63	0.01
	Degree of satisfaction with income (X_20_)	0.294	0.47	0.101	0.813
(5) Institutions	Policies that encourage migration to urban areas (*X*_21_)			0.123	0.79
	“One family, one house” policy (*X*_22_)			0.129	0.763
	Homestead and contracted land subsidy policy (*X*_23_)			1.63	0.002
(6) Constraints	Income constraint (*X*_24_)			−0.807	0.008
	Employment constraint (*X*_25_)			−0.407	0.346
	Housing constraint (*X*_26_)			−0.736	0.441
	Social security (*X*_27_)			−0.865	0.484
	Other constraints (*X*_28_)			−1.271	0.033
Constant		0.354	0.087	−3.908	0.007
Chi-square		10.113 (Sig. = 0.006)		15.985 (Sig. = 0.001)	
Cox & Snell *R*^2^		0.053		0.107	
Nagelkerke *R*^2^		0.077		0.149	
Sample size		157		157	

**Table 4:** Cluster analysis results of migration willingness of farmers classified according to individual characteristics.

Variable	Youth Group		Middle-Aged/Aged Group		Low Education Group		High Education Group		Agricultural Production Group		Non-Agricultural Production Group	
	B	Sig.	B	Sig.	B	Sig.	B	Sig.	B	Sig.	B	Sig.
*X*_23_	1.32	0.05	1.24	0.07	1.18	0.03	0.99	0.25	1.15	0.02	0.99	0.09
*X*_24_	−0.33	0.48	−0.46	0.39	−1.66	0.08	−0.26	0.61	−0.39	0.43	−1.47	0.21
*X*_28_	−0.79	0.48	−1.32	0.09	−1.10	0.09	−0.81	0.48	−1.06	0.14	−1.59	0.28
Constant	−1.25	0.08	−2.17	0.06	−2.03	0.07	−0.76	0.02	−1.87	0.07	−0.49	0.20
Chi-square	4.51(Sig = 0.09)		14.07(Sig = 0.08)		3.81(Sig = 0.05)		5.54(Sig = 0.06)		4.79(Sig = 0.03)		5.53(Sig = 0.05)	
Cox & Snell *R*^2^	0.10		0.09		0.06		0.17		0.06		0.17	
Nagelkerke *R*^2^	0.14		0.14		0.09		0.24		0.09		0.24	
Sample size	43		147		146		44		161		29	

**Table 5:** Cluster analysis results of migration willingness of farmers classified according to family economic basis.

Variable	Group without Non-Agricultural Workers		Group with Non-Agricultural Workers		Low Income Group		High Income Group	
	B	Sig.	B	Sig.	B	Sig.	B	Sig.
*X*_23_	1.70	0.01	1.12	0.1	1.6	0.02	0.87	0.1
*X*_24_	−0.62	0.26	−1.21	0.06	−0.90	0.08	−1.16	0.07
*X*_28_	−0.58	0.54	−2.43	0.03	−1.18	0.10	−1.64	0.09
Constant	−3.53	0.00	−0.68	0.02	−3.01	0.00	−1.01	0.04
Chi-square	6.888(Sig. = 0.009)		7.820(Sig. = 0.045)		5.723(Sig. = 0.017)		7.820(Sig. = 0.451)	
Cox & Snell *R*^2^	0.16		0.12		0.11		0.09	
Nagelkerke *R*^2^	0.25		0.17		0.16		0.12	
Sample size	73		117		104		86	
